# Effectivity of One Session Charcoal Hemoperfusion Treatment in Severe Carbamazepine Poisoning

**DOI:** 10.5812/ircmj.7516

**Published:** 2013-08-05

**Authors:** Yasemin Isik, Lokman Soyoral, Sevdegul Karadas, Habib Emre, Muhammed Bilal Cegin, Ugur Goktas

**Affiliations:** 1 Department of Anesthesiology and Intensive Care, Medical Faculty, Yuzuncu Yil University, Van, Turkey; 2 Department of Anesthesiology and Intensive Care, State Hospital, Van, Turkey; 3 Department of EmergencFaculty, Yuzuncu Yil University, Van, Turkey; 4 Department of Nephrology, Medical Faculty, Yuzuncu Yil University, Van, Turkey; 5 Department of Anesthesiology and Intensive Care, Medical Faculty, Yuzuncu Yil University, Van, Turkey

**Keywords:** Carbamazepine, Toxicity, Hemoperfusion

## Abstract

A carbamazepine intoxication with suicide attempt is a relatively common clinical problem that presenting with coma, respiratory depression, arrhythmia, hemodynamic instability and even death. We report a case of severe carbamazepine poisoning that was successfully treated with one session charcoal hemoperfusion. On admission, the patient was comatose and required ventilator support. Hemoperfusion with coated activated charcoal successfully decreased the serum carbamazepine concentration from 45 µg mL^−1^ to 21 µg mL^−1^ within 2 h, with subsequent clinical improvement.

## 1. Introduction

A carbamazepine (CBZ) is a widely prescribed drugs that using in the treatment of epilepsy, neuralgic pain syndromes and certain affective disorders. A carbamazepine intoxication with suicide attempt is a relatively common clinical problem that presenting with coma, respiratory depression, arrhythmia, hemodynamic instability and even death (1-3). The mortality rate due to / CBZ toxicity is around 13% (3). The adult therapeutic CBZ plasma level is between 4 and 12 µg mL^−1^ (4). Several therapeutic interventions including charcoal hemoperfusion (HP), hemodialysis (HD), hemodiafiltration and plasma exchange have been performed for the treatment of acute CBZ intoxication (5, 6). However, overall multiple-dose activated charcoal HP seems to the most effective and popular treatment option (7, 8). We report a case of severe carbamazepine poisoning who was treated with one session charcoal hemoperfusion.

## 2. Case Report

A 19-year-old girl was brought to an emergency room 4 hours after ingestion of 100 tablets CBZ (each CBZ tablet was 400 mg). She was transferred to intensive care unit 6 hours after the ingestion. On admission, in the physical examination, she was unconscious but responsive to pain stimuli, Glasgow coma score (GCS): 8, her blood pressure: 110/55 mmHg, pulses: 120/min, temperature: 36.7°C and respiratory rate: 20/min. The breath sounds were coarse and heart sounds were rhythmic and tachycardic. Other system examinations were normal. A Gastric lavage was performed and an activated charcoal was given once before arrival to the hospital. Isotonic solution (%0.9 NaCl) was administered IV to the patient. On admission, her CBZ level was 53 µg mL ^−1 ^. Laboratory tests during admission and follow-ups are shown in Table 1. On the eight hours after ingestion evaluation, she was still comatose and was intubated. Patient underwent ventilation with Synchronized intermittent mandatory ventilation (SIMV) mode: pressure control ventilation, 12-14 cm H _2 _O; inspiratory time, 0.8 s; triggering sensitivity, 0.6 L min ^−1 ^; positive endexpiratory pressure, 4 cm H _2 _O; and FiO _2 _:40-50%. The hemoperfusion cartridge was not readily available in our instution at the beginning, so we initially administered hemodiafiltration for 6 hours period. After providing hemoperfusion cartridge, 4 h of charcoal-HP was administered. The blood ﬂow rate was 200 to 300 mL min ^−1 ^and the dialysate flow rate: 300-500 mL min ^−1 ^. These rates varied with the hemodynamic chancing of the patient and regarding blood gases analyses to eliminate more drugs from blood. Heparin anticoagulation was initiated with a 1000 U bolus, followed by a continuous infusion of 750 U h ^−1 ^, and was adjusted as needed to achieve a partial thromboplastin time of 80 to 100 seconds. Repeated administration was not needed after 4 h charcoal-HP. The blood CBZ level just after the procedure was 20 µg mL ^−1 ^, and it declined to 8 µg mL ^−1 ^at 72 hours of the admission. The follow-up CBZ levels are summarized in Table 2. At the end of the procedure, she was extubated. She was discharged from hospital on the fourth day. 

**Table 1.** Patients’ Laboratory Tests

	1. day	2. day	3. day	4. day
**Glucose(mg/dL)**	138	184	112	107
**Creatinine(mg/dL)**	0,7	0,3	0,5	0,5
**ALT(U/L)**	16	18	16	20
**Haemoglobin(g/dL)**	12	8,4	9,9	10,2
**Platelet (103)**	186	119	97	127
**INR**	1,48	2,37	1,6	1,2
**Potassium(mmol/L)**	3,9	2,4	2,7	4
**Calcium(mg/dL)**	8,1	7,9	8,3	8,2

**Table 2.** Blood Carbamazepine Levels of the Patients Before and After Treatment

Before HDF procedure	After HDF procedure	Before Charcoal-HP procedure	1.h	2. h	4. h	12.h	24.h	48.h	3.day
**53**	45	45	19	21	20	20	19	19	8

## 3. Discussion

CBZ intoxication can cause seizures, coma, arrhythmias and even death (1-3). There is a complex distribution and metabolism of CBZ. CBZ is slowly absorbed from gastrointestinal system and also has inhibitory action on bowel motility. Thus the peak CBZ level is accomplished within 10 to 30 hours following oral ingestion. The oral CBZ absorption rate is around 70-95%, the serum CBZ-protein binding rate is 80–85% with a moderately large volume of distribution. During acute intoxication, CBZ pharmacokinetic features can interfere its rapid elimination from the body (5, 9). The multiple dose administration of activated charcoal increase CBZ clearance from the body by interfering with entero-enteric circulation of CBZ. If serum CBZ concentration is greater than 40 mcg/mL, serious complications including coma, respiratory depression, seizures, and ventricular arrhythmias can occur (1, 2). The measurement of the primary metabolite CBZ-10, 11-epoxide is not readily available in our institution, so we measured total CBZ plasma concentration. In our case, the CBZ level was 53 µg mL^-1^ on the admission. She had sinus tachycardia and was intubated due to development of respiratory depression. The hemoperfusion cartridge was not readily available in our institution; therefore we applied four hours hemodiafiltration. After four hours hemodiafiltration, the CBZ level was decreased to 45 µg mL^-1^. Then four hours-charcoal hemoperfusion was administered. A charcoal HP was preferred as it has been the most commonly recommended intervention for severe acute CBZ. In the previous study, hemoperfusion was shown to be superior to hemodialysis (10). Choarcoal-HP can reduce serum CBZ concentrations by 25 to 50% (8, 10-12). In severe cases, the artificial ventilation may be needed for several days after the hemoperfusion (10-12). The persistence of neurologic effects may be related to absorption of the medication from the gut.(10, 12) Our patient was extubated after one session hemoperfusion and no neurological complication was observed. CBZ level was not diminished after 1 hour that might be related to reduced charcoal absorptive capacity after the first hour with prolonged treatment, reflecting cartridge saturation.

The hemoperfusion-related side effects are hypocalcaemia, thrombocytopenia, coagulopathies, and hypothermia. The hypocalcaemia, hypokalemia, thrombocytopenia and coagulopathy were seen in our patient. The hypocalcaemia and hypokalemia were replaced but treatment did not need for thrombocytopenia and coagulopathy. However, all the parameters were normalized 4 days following the treatment (Table 1). 

## 4. Conclusions

We conclude that one session charcoal-hemoperfusion is a safe and effective alternative treatment for life-threatening severe carbamazepine intoxication. Complications by charcoal-hemoperfusion should be considered. Charcoal-hemoperfusion is preferred to prevent further seizures, shorten mechanical ventilation time and intensive care unit time, and decrease risk of infectious or neurological complications.
